# Construction of α-MnO_2_ on Carbon Fibers Modified with Carbon Nanotubes for Ultrafast Flexible Supercapacitors in Ionic Liquid Electrolytes with Wide Voltage Windows

**DOI:** 10.3390/nano12122020

**Published:** 2022-06-11

**Authors:** Mai Li, Kailan Zhu, Hanxue Zhao, Zheyi Meng, Chunrui Wang, Paul K. Chu

**Affiliations:** 1 College of Science, Donghua University, Shanghai 201620, China; 2202257@mail.dhu.edu.cn (K.Z.); z18238630680@163.com (H.Z.); crwang@dhu.edu.cn (C.W.); 2 State Key Laboratory for Modification of Chemical Fibers and Polymer Materials, College of Materials Science, Donghua University, Shanghai 201620, China; 3 Department of Physics, City University of Hong Kong, Tat Chee Avenue, Kowloon, Hong Kong 999077, China; paul.chu@cityu.edu.hk

**Keywords:** carbon nanotubes, composite materials, manganese dioxide, flexible supercapacitors, ionic electrolytes

## Abstract

In this study, α-MnO_2_ and Fe_2_O_3_ nanomaterials are prepared on a carbon fiber modified with carbon nanotubes to produce the nonbinder core–shell positive (α-MnO_2_@CNTs/CC) and negative (Fe_2_O_3_@CNTs/CC) electrodes that can be operated in a wide voltage window in ultrafast asymmetrical flexible supercapacitors. MnO_2_ and Fe_2_O_3_ have attracted wide research interests as electrode materials in energy storage applications because of the abundant natural resources, high theoretical specific capacities, environmental friendliness, and low cost. The electrochemical performance of each electrode is assessed in 1 M Na_2_SO_4_ and the energy storage properties of the supercapacitors consisting of the two composite electrodes are determined in Na_2_SO_4_ and EMImBF4 electrolytes in the 2 V and 4 V windows. The 2 V supercapacitor can withstand a large scanning rate of 5000 mV S^−1^ without obvious changes in the cyclic voltammetry (CV) curves, besides showing a maximum energy density of 57.29 Wh kg^−1^ at a power density of 833.35 W kg^−1^. Furthermore, the supercapacitor retains 87.06% of the capacity after 20,000 galvanostatic charging and discharging (GCD) cycles. The 4 V flexible supercapacitor shows a discharging time of 1260 s and specific capacitance of 124.8 F g^−1^ at a current of 0.5 mA and retains 87.77% of the initial specific capacitance after 5000 GCD cycles. The mechanical robustness and practicality are demonstrated by physical bending and the powering of LED arrays. In addition, the contributions of the active materials to the capacitive properties and the underlying mechanisms are explored and discussed

## 1. Introduction

Supercapacitors are energy storage devices based on both physical adsorption and desorption, as well as electrochemical reactions, and have attracted extensive attention because of advantages such as the fast charging and discharging capability, high power density, robust cycling stability, and environmental friendliness [1,2]. The electrode materials, structure, and electrolyte determine the properties of the supercapacitors [3,4,5]. With regard to the electrode materials, various forms of carbonaceous materials, conductive polymers, and transition metal oxides are commonly used as the active materials in supercapacitors [6,7,8,9]. In particular, manganese oxide (MnO_2_) originated in the battery industry and has been widely used in the area of batteries and supercapacitors that promote our in-depth understanding and research on this material [10]. It is interesting because of the natural abundance, environmental friendliness, high theoretical specific capacity, low cost, and compatibility with other materials in energy storage applications [10,11,12]. However, MnO_2_ electrodes are prone to severe agglomeration and the volume expansion during electrochemical cycling and the rate capability are low because of the poor conductivity [13,14,15]. Therefore, the optimization of MnO_2_-based materials is crucial for improving the properties and the power density while maintaining the good energy density.

The integration of MnO_2_-based electrodes with carbonaceous materials such as graphene and carbon nanotubes can improve the properties of supercapacitors [16,17,18,19]. Carbon nanotubes can improve the conductivity, specific capacitance, and ion migration efficiency of the active materials, and increase the mechanical adhesion strength between the active materials and the substrate [20,21,22,23]. For example, a mild one-pot reaction has been demonstrated to synthesize the MnO_2_/CNTs composite which has a capacity of 201 F g^−1^ and does not show obvious decay after 10,000 cycles at a current density of 1 A g^−1^ [17]. The capacity of the MnO_2_/CNTs-CNFS composite electrode prepared by Wang et al. is 374 F g^−1^ and 94% of its capacitance is retained after 1000 cycles [24]. The needle-shaped MnO_2_-CNTs-CFC composite fabricated by Li et al. by the two-step electrophoretic deposition shows a specific capacity of 381.74 F g^−1^ and an 85% capacitance retention after 1000 cycles [18]. Therefore, the MnO_2_@CNTs composite is a potential electrode for supercapacitors. Although MnO_2_@CNTs have been studied, there are few reports on the preparation of this structure on flexible carbon fiber cloth, and the research of asymmetric flexible supercapacitors prepared based on this structure has just begun. Furthermore, a better understanding of the mechanism pertaining to the electrochemical kinetics and effects of different electrolytes on the electrochemical characteristics of MnO_2_@CNTs-based supercapacitors are required in the development of high-performance electrodes [5].

**Figure 1:**
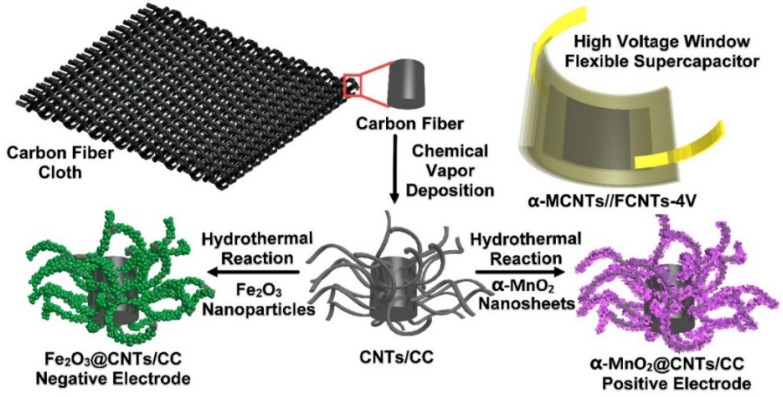
Schematic illustration of the fabrication of the electrodes and flexible supercapacitor with a large voltage window comprising the α-MnO_2_@CNTs/CC positive electrode and Fe_2_O_3_@CNTs/CC negative electrode.

In this study, a novel structure composed of α-phase ultrathin MnO_2_ films prepared on a piece of carbon fiber cloth modified with carbon nanotubes (CNTs/CC) is prepared. The CNTs offer advantages such as a large surface area, efficient ion diffusion, large active substance loading, and excellent substrate conductivity to improve the rate as well as the energy and power densities of the electrode. The α-MnO_2_@CNTs/CC positive electrode and Fe_2_O_3_@CNTs/CC negative electrodes are prepared (Figure 1) [25] and the electrochemical properties of the two electrodes are determined in 1 M Na_2_SO_4_. An asymmetrical supercapacitor (ASCs) consisting of the α-MnO_2_@CNTs/CC as the positive electrode, Fe_2_O_3_@CNTs/CC as the negative electrode, and 1 M Na_2_SO_4_ as the electrolyte is assembled to provide a 2 V window (α-MCNTs//FCNTs-2V). In order to demonstrate the practicality, a flexible asymmetrical supercapacitor (FASC) with the same electrode combination but different ionic electrolyte of ionic liquid is constructed for the 4 V window (α-MCNTs//FCNTs-4V). The electrochemical performance and mechanism of the electrodes and devices are investigated and discussed.

## 2. Materials and Methods

### 2.1. Materials Preparation

The fabrication process and structure of the α-MnO_2_@CNTs/CC positive electrode, Fe_2_O_3_@CNTs/CC negative electrode, and α-MCNTs//FCNTs-4V flexible supercapacitor are shown in Figure 1. The CNTs were prepared on a carbon fiber cloth by chemical vapor deposition and then the MnO_2_ and Fe_2_O_3_ were fabricated on the CNTs hydrothermally. The chemicals used were analytical grade.

The carbon fiber cloth was cut into 1 × 1 cm^2^ pieces and cleaned with a nitrogen plasma (200 W) for 10 min in vacuum. Fe as the seed for carbon nanotubes was electrodeposited on the carbon fiber cloth in 20 mM Fe(NO_3_)_2_ at a current density of 3 mA and CNTs/CC was then prepared by chemical vapor deposition under nitrogen and methane at 800 °C for 2 h.

The α-MnO_2_ film was deposited on CNTs/CC hydrothermally. Potassium permanganate (0.158 g) was dissolved in 20 mL of deionized water and the clean 1 × 1 cm^2^ CNTs/CC was placed in the solution in a container made of Teflon. After sealing, the container was placed in a reactor and heated to 180 °C for 12 h. After natural cooling to room temperature, the product was rinsed with deionized water several times and dried at 70 °C for 12 h. Fe_2_O_3_ was prepared fabricated on CNTs/CC by the same method a the α-MnO_2_ film except that 2 mmol Fe(NO_3_)_2_ and 15 mmol urea were used in the precursor solution and the hydrothermal reaction proceeded at 120 °C for 8 h. The α-MnO_2_/CC and Fe_2_O_3_/CC electrodes were produced by the same method by direct deposition of the active materials on the carbon fiber cloth.

### 2.2. Preparation of Asymmetrical Supercapacitors

Asymmetrical supercapacitors consisting of the α-MnO_2_-based positive electrode, Fe_2_O_3_-based negative electrode, and the nonwoven fabric separator were prepared. An aqueous electrolyte was used in the 2 V supercapacitor of α-MCNTs//FCNTs-2V (α-MnO_2_@CNTs/CC as the positive electrode and Fe_2_O_3_@CNTs/CC as the negative electrode). The pretreatment involved soaking the separator and electrodes in 1 M NaSO_4_ for 10 min and the separator and electrodes were assembled into a CR2032 shell as a sandwiched structure. The same method used for α-MCNTs//FCNTs-2V was adopted to fabricate α-M//F-2V (α-MnO_2_/CC as the positive electrode and Fe_2_O_3_/CC as the negative electrode) and CNTs//CNTs (CNTs/CC as both the positive and negative electrode) for comparison. To prepare the ultrafast α-MCNTs//FCNTs-4V flexible supercapacitor with a large voltage window, the nonwoven fabric separator and electrodes were dipped in an ionic liquid (1-ethyl-3-methylimidazolium tetrafluoroborate (EMImBF4)) for 10 min. The positive and negative electrodes were separated and encapsulated with the polyimide tape. Again, the same technique employed to prepare α-MCNTs//FCNTs-4V was implemented to fabricate α-M//F-4V (α-MnO_2_/CC as the positive electrode and Fe_2_O_3_/CC as the negative electrode) for comparison.

### 2.3. Materials Characterization

The morphology and microstructure were examined by scanning electron microscopy (SEM) on the 7500F (JEOL, Tokyo, Japan) and the elemental states and composition were determined by X-ray photoelectron spectroscopy (XPS) using the ESCALAB-250 (Thermo Fisher Scientific, Waltham, MA, USA). The crystal structure was determined by X-ray diffraction (XRD, D/max-2550, Rigaku, Tokyo, Japan) and the morphology and lattice of α-MnO_2_@CNTs and Fe_2_O_3_@CNTs were examined by the transmission electron microscopy (TEM) on the JEOL2100 (JEOL, Tokyo, Japan) at 200 kV.

### 2.4. Electrochemical Measurements

The electrochemical assessment was carried out on the CHI660E electrochemical workstation. In the configuration with 1.0 M Na_2_SO_4_ electrolyte, CC, CNTs/CC, and α-MnO_2_@CNTs/CC or Fe_2_O_3_@CNTs/CC were the working electrodes, whereas a saturated calomel was the reference electrode and platinum wire was the counter electrode. Galvanostatic charging/discharging (GCD), cyclic voltammetry (CV), and electrochemical impedance spectroscopy (EIS) were performed in addition to self-discharging and monitoring of the leakage currents. For the asymmetrical supercapacitor containing 1.0 M Na_2_SO_4_ and ionic liquid as the electrolytes, respectively, GCD, CV, and EIS were performed on the electrochemical workstation and long-time cycling was carried out on the LAND supercapacitor testing system.

The specific capacitance of the composite electrodes $C_{s}\;$ was calculated by Equation (1) and the energy density (*E*) and power density (*P*) were calculated by Equations (2) and (3), respectively [26,27]:

(1) $$C_{s}=\frac{I\times \Delta t}{m\times \Delta V}\;,$$

(2) $$E=\frac{C\times {\left(\Delta V\right)}^{2}}{2\times 3.6}\;,\;\mathrm{and}\;$$

(3) $$P=\frac{E\times 3600}{\Delta t}.$$

Here, $C_{s}$ (F g^−1^) is the specific capacitance, I(A) represents the current or current density during the GCD process, m(g) is the mass of active materials on the surface of the electrodes, Δt(s) denotes the time in the discharging process of the GCD test, and ΔV(V) stands for the voltage window of the electrodes or asymmetrical supercapacitor in the GCD test.

## 3. Results

### 3.1. Materials Characterization

**Figure 2:**
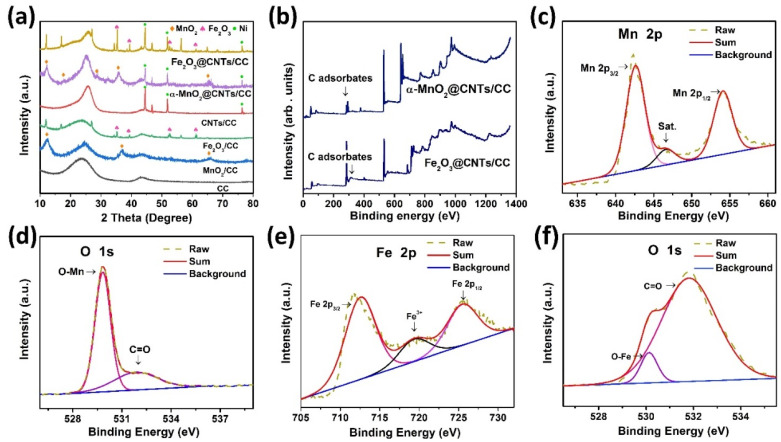
(**a**) XRD spectra of α-MnO_2_/CC, Fe_2_O_3_/CC, CNTs/CC, α-MnO_2_@CNTs/CC, and Fe_2_O_3_@CNTs/CC; (**b**) Survey XPS spectra of the α-MnO_2_@CNTs/CC and Fe_2_O_3_@CNTs/CC composite electrodes; High-resolution XPS spectra: (**c**) Mn 2*p* of α-MnO_2_@CNTs/CC, (**d**) O 1*s* of α-MnO_2_@CNTs/CC, (**e**) Fe 2*p* of Fe_2_O_3_/CNTs/CC, and (**f**) O 1*s* of Fe_2_O_3_/CNTs/CC.

Figure 2a exhibits the XRD patterns of α-MnO_2_/CC, Fe_2_O_3_/CC, CNTs/CC, α-MnO_2_@CNTs/CC, and Fe_2_O_3_@CNTs/CC. The peak at 25.74° represents carbon, and according to PDF#44-0141, the peaks at 12.78°, 18.11°, 28.84°, 36.70°, and 66.69° stem from α-MnO_2_ matching α-MnO_2_/CC and α-MnO_2_@CNTs/CC, as shown in Figure 2a [28]. The XRD patterns of the Fe_2_O_3_ deposited on the carbon fiber cloth and the CNTs-modified carbon fiber cloth are shown in the yellow and green curves in Figure 2a, respectively. The two samples exhibit peaks of Fe_2_O_3_ at 35.61°, 40.85°, 54.09°, and 62.45°, corresponding to the (110), (113), (116), and (214) planes of α-Fe_2_O_3_ (PDF#33–0664), respectively [29]. The XPS survey spectrum of Figure 2b shows the existence of C, O, and Mn in α-MnO_2_@CNTs/CC and C, O, and Fe in Fe_2_O_3_@CNTs/CC. Figure 2c shows the Mn 2*p* spectrum of α-MnO_2_@CNTs/CC, revealing peaks at 654.2 eV for Mn 2*p*_1/2_ and 642.5 eV for Mn 2*p*_3/2_ of MnO_2_. The satellite peak (sat) is related to α-MnO_2_, consistent with previous studies and further confirming the formation of MnO_2_ [28]. The H-O-H (531.9 eV) and Mn-O-H (530.2 eV) peaks in Figure 2d of α-MnO_2_@CNTs/CC are attributed to the hydroxyl groups and absorbed water, respectively [30,31,32]. The Fe 2*p* peaks at 724.58 eV and 710.86 eV in Figure 2e are the Fe 2p peaks of Fe_2_O_3_@TiN/CC representing Fe 2*p*_1/2_ and Fe 2*p*_3/2_ in α-Fe_2_O_3_ [28]. As shown in Figure 2f, the peaks at 530.0, 531.4, and 532.5 eV of Fe_2_O_3_@CNTs/CC arise from O^2−^, OH^−^, and O-C=O, respectively [25], and that at 532.6 eV corresponds to the H-O-H of the adsorbed water molecules [33]. The peak at 531.4 eV reflects the O-H of the surface or the internal hydroxyl groups and the chemically adsorbed oxygen [34], and that at 530.0 eV stems from the O^2−^ in Fe_2_O_3_ [35].

**Figure 3:**
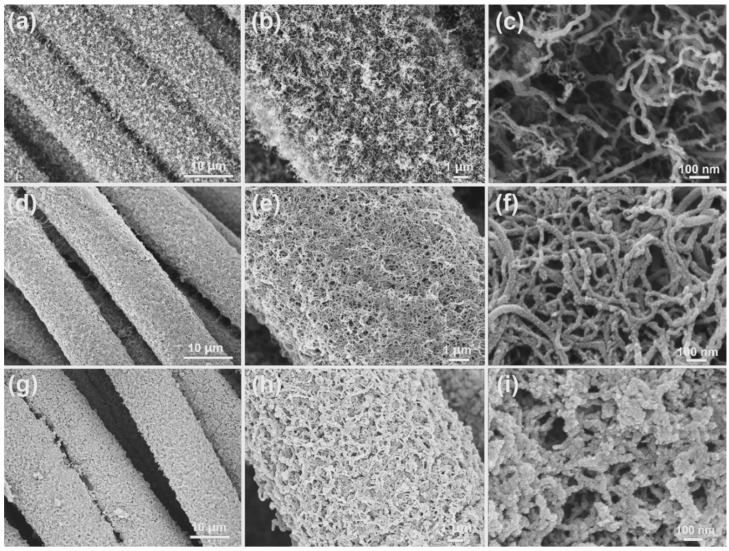
SEM images of (**a**–**c**) CNTs/CC, (**d**–**f**) α-MnO_2_@CNTs/CC, and (**g**–**i**) Fe_2_O_3_@CNTs/CC at different magnifications.

Figure 3a–i display the SEM images of CNTs/CC, α-MnO_2_@CNTs/CC, and Fe_2_O_3_@CNTs/CC at different magnifications. Carbon nanotubes with a diameter of 10–20 nm are observed on the carbon fiber cloth (Figure 3a–c) to produce a conductive network for the α-MnO_2_ nanosheets and Fe_2_O_3_ nanodots. The carbon nanotubes are connected in a staggered and dense way to promote the transfer of electrons, ion extraction and insertion, and the effective area for the active materials. As shown in Figure 3d–f, the CNTs form a 3D porous skeleton and provide abundant nucleation sites for uniform MnO_2_ deposition, while preventing the agglomeration of the active substances during the electrochemical reaction [27]. Figure 3f shows many micropores in the α-MnO_2_@CNTs/CC electrode for enhanced electrolyte circulation in the electrode and reduced contact impedance between the electrode and electrolyte. According to the SEM images in Figure 3g–i, nanoscale Fe_2_O_3_ is deposited on CNTs/CC to form the Fe_2_O_3_@CNTs/CC negative electrode and the morphological and structural characteristics of the negative electrode are similar to those of the positive electrode. In the ionic electrolyte, the α-MnO_2_@CNTs/CC positive electrode and the Fe_2_O_3_@CNTs/CC electrode broaden the voltage window of the flexible supercapacitor to 4 V, thereby improving the power density without compromising the energy density of the device [36].

**Figure 4:**
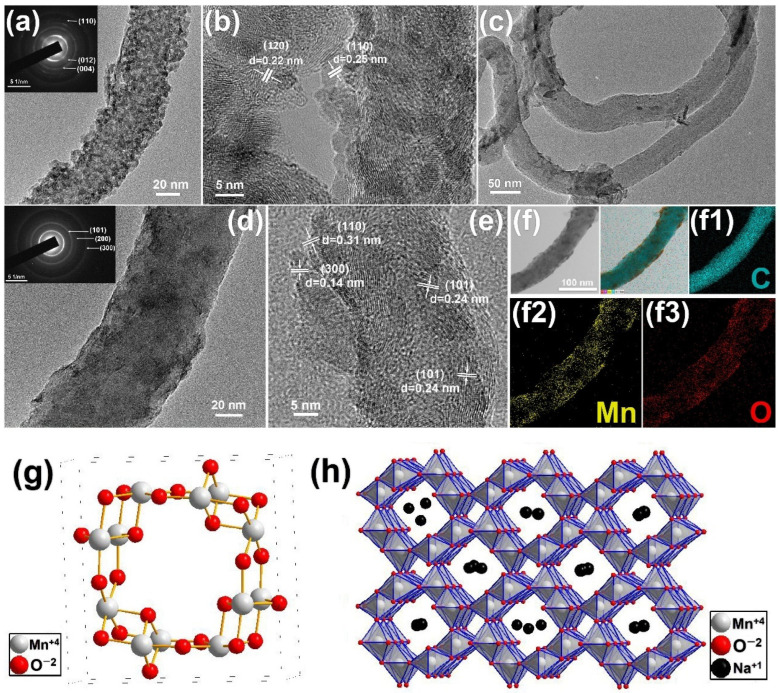
(**a**) TEM image and SAED pattern and (**b**) HR−TEM image of the Fe_2_O_3_@CNTs core−shell structure; (**c**,**d**) TEM image and SAED pattern, (**e**) HR−TEM image and (**f**) The STEM image (left), total EDS element mapping (right) and the corresponding EDS mappings of C (f1), Mn (f2), O (f3) elements of α-MnO_2_@CNTs core−shell structure; (**g**) the unit cell of α-MnO_2_; (**h**) crystal structure of α-MnO_2_ with one-dimensional pore morphology and schematic diagram of sodium ion adsorption and embedding in pore structure.

The α-MnO_2_@CNTs and Fe_2_O_3_@CNTs core–shell structures were stripped from the composite electrodes ultrasonically to analyze by TEM. As shown in Figure 4a, the Fe_2_O_3_ nanoparticles are densely and uniformly deposited on the carbon nanotubes, forming the Fe_2_O_3_@CNTs nanocomposite with a diameter of about 40 nm. The high-resolution image in Figure 4b indicates lattice spacings of 0.22 nm and 0.25 nm matching the (120) and (110) planes of Fe_2_O_3_ [37]. Figure 4c,d show that α-MnO_2_@CNTs composite has a diameter of about 50 nm, which is different from that of Fe_2_O_3_@CNTs, but the general morphology of the carbon nanotubes is preserved. The high-resolution image of the α-MnO_2_@CNTs core–shell structure in Figure 4e reveals lattice spacings of 0.14 nm, 0.31 nm, and 0.24 nm corresponding to the (300), (101), and (110) planes of α-MnO_2_, respectively [27,38]. The core–shell structure comprising the MnO_2_ film and CNTs is confirmed by the elemental maps of α-MnO_2_@CNTs in Figure 4f and the elemental distributions of C, Mn, and O in Figure 4(f1–f3), indicating that large amounts of Mn and O are uniformly distributed on the CNTs [28].

Figure 4g depicts the unit cell of α-MnO_2_, and the basic structure of the unit cell is the eight surfaces [MnO_6_] formed by the manganese atoms surrounded with six oxygen atoms, and the common edge of the eight surfaces forms a double chain along the c axis, as shown in the crystal structure of Figure 4f [39]. The eight surfaces of these double chains share the apex with the neighboring double chains to form a [2 × 2] tunnel, and this large tunnel can accept cations with a radius of around 0.15 nm, such as Ba^2+^, K^+^, Pb^2+^, Na^+^, and NH_4_^+^, as well as H_2_O molecules [40]. In the charge and discharge process of the α-MnO_2_ electrode material, the conversion of manganese atoms is between +3 valence and +4 valence, forming two energy storage mechanisms: one is ion adsorption and desorption on α-MnO_2_ surface [41]:

(4) $${\left(MnO_{2}\right)}_{surface}+Na^{+}+e^{-}↔{\left(MnO_{2}^{-}Na^{+}\right)}_{surface}$$

the other is ion insertion and extraction in the inter tunnel of α-MnO_2_:

(5) $$MnO_{2}+Na^{+}+e^{-}↔\left(MnOONa\right)$$

The first energy storage mechanism comes from the high specific surface area of α-MnO_2_; meanwhile, the second is based on the effective structural tunnel of manganese dioxide.

### 3.2. Electrochemical Properties of The Fabricated Electrodes

**Figure 5:**
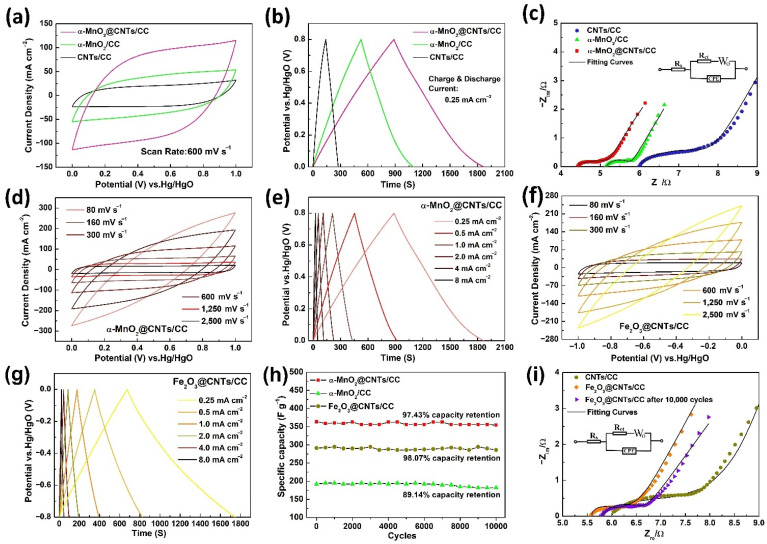
(**a**) CV curves, (**b**) GCD curves, and (**c**) Nyquist plots of CNTs/CC, α-MnO_2_/CC, and α-MnO_2_@CNTs/CC; Electrochemical properties of α-MnO_2_@CNTs/CC: (**d**) CV curves, (**e**) GCD curves; Electrochemical properties of Fe_2_O_3_@CNTs/CC: (**f**) CV curves, (**g**) GCD curves; (**h**) 10,000 cycling evaluation, and (**i**) Nyquist plots of Fe_2_O_3_@CNTs/CC before and after 10,000 cycles.

As shown in Figure 5a–c and Figure S1, the electrochemical properties of CNTs/CC, α-MnO_2_/CC, and α-MnO_2_@CNTs/CC are similar, but α-MnO_2_@CNTs/CC shows a longer discharging time, a larger CV area, a smaller contact resistance, and a higher ion diffusion efficiency than CNTs/CC and α-MnO_2_/CC. According to Figure S3, Table S1, and Equation (1), the MnO_2_ loading of α-MnO_2_ on the carbon fiber cloth is 0.55 mg per cm^2^, and that of α-MnO_2_ on the carbon fiber cloth with CNTs is 0.73 mg per cm^2^. The specific capacity of α-MnO_2_@CNTs/CC is 367.44 F g^−1^ at a current density of 2 mA cm^−2^, which is superior to that of 188.55 F g^−1^ of α-MnO_2_/CC. Hence, the carbon nanotubes play a significant role in improving the electrochemical activity. The characteristics of the three electrodes shown in Figure 5a,b are consistent with the Nyquist curves in Figure 5c, and the detailed fits of the Nyquist curves are shown in Table S2. As shown in Figure 5d, the cyclic voltammograms of α-MnO_2_@CNTs/CC between 80 mV s^−1^ and 2500 mV s^−1^ show the typical rectangular shape without obvious deformation, even at a large scanning rate [27]. As shown in Figure 5e, the GCD curves of α-MnO_2_@CNTs/CC from 0.25 mA cm^−2^ to 8.0 mA cm^−2^ exhibit a similar shape of an isosceles triangle, consistent with the characteristics of the pseudocapacitance. The corresponding specific capacitances are listed in Table S1.

To assess the matching between the negative and positive electrodes, electrochemical tests are performed on Fe_2_O_3_@CNTs/CC, as shown in Figure 5f–i. Figure 5f shows the CV results at scanning rates between 80 mV s^−1^ and 2500 mV s^−1^ in the voltage window from 0 to −1 V. The results reflect the abundant active sites boding well for rapid ion extraction and insertion. GCD curves are acquired from Fe_2_O_3_@CNTs/CC from 0.25 mA cm^−2^ to 8 mA cm^−2^ (Figure 5g), and the time duration of GCD increases with the decreasing current densities. The CV and GCD curves of CNTs/CC are shown in Figure S2 for comparison. At a current density of 0.5 mA cm^−2^, CNTs/CC shows a discharging time of 260.1 s, whereas Fe_2_O_3_@CNTs/CC shows a discharging time of 958 s, confirming that the Fe_2_O_3_@CNTs composite shows enhanced performance. Figure 5h exhibits the stability curves of the α-MnO_2_@CNTs/CC, α-MnO_2_/CC, and Fe_2_O_3_@CNTs/CC electrodes for 10,000 GCD cycles. The specific capacitance of α-MnO_2_@CNTs/CC is 361.8 F g^−1^ at a current density of 3 mA cm^−2^, and after 10,000 cycles, the attenuation is only 2.6% to 350.9 F g^−1^, which is better than the observed 89.36% of α-MnO_2_/CC. The impedance data of the Fe_2_O_3_@CNTs/CC negative electrode before and after 10,000 cycles in Figure 5i are consistent with the cycling tests showing 93.54% capacity retention. The detailed analysis and fits of the Nyquist curves in Figure 5i are listed in Table S2.

**Figure 6:**
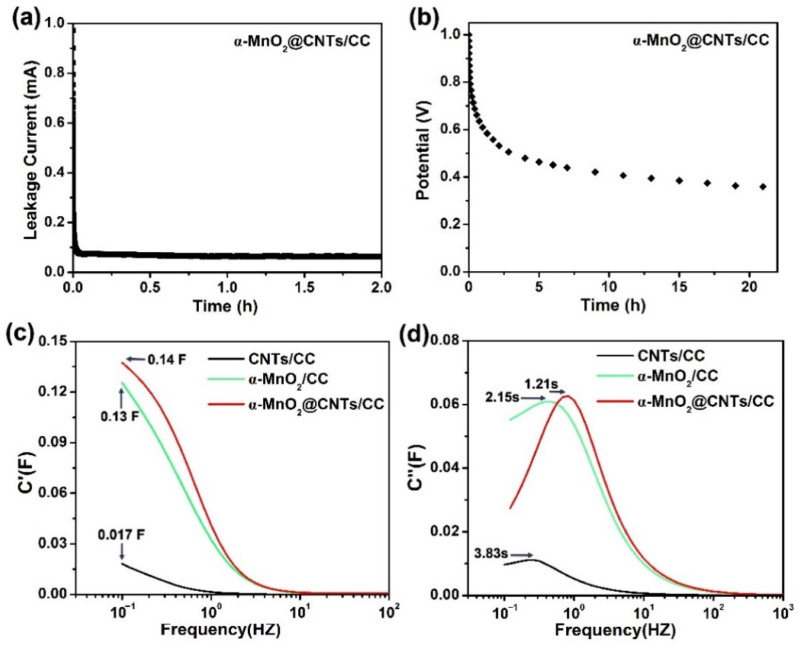
(**a**) Self-discharging and (**b**) leakage currents in self-discharging of the α-MnO_2_@CNTs/CC electrode in 1 M Na_2_SO_4_; Evolution of the (**c**) real part and (**d**) imaginary capacitance vs. frequency for CNTs/CC, α-MnO_2_/CC, and α-MnO_2_@CNTs/CC electrodes in 1 M Na_2_SO_4_.

The leakage current and self-discharging are important parameters. To measure the leakage current, the composite electrode is the working electrode in the three-electrode system, and pulses of 1 V at 2 mA are applied. The leakage currents of the α-MnO_2_@CNTs/CC electrode versus time are shown in Figure 6a, disclosing a quick drop to 0.063 mA in a short time before stabilization in the next 2 h. The leakage current of the positive electrode is less than 0.34 mA of the 0.086 mA of 3D-NCS-3//N-rGO [42], Ni-Mn LDH/MnO_2_ [43], and 0.08 mA of a-NENCs [44]. The small leakage current indicates an insignificant electrolyte diffusion on the electrode surface and fewer side reactions caused by impurities on the electrode [45]. The self-discharging process of the α-MnO_2_@CNTs/CC electrode shown in Figure 6b is monitored by measuring the potentials of the electrode under open circuit conditions for 21 h [46]. Owing to the leakage current, the potentials of the electrode decrease gradually with time and the potential drop is significant in the first 2–3 h before moderation in the subsequent 10 hours. All in all, the potential time curves in Figure 6b show a stable output potential of 467 mV after 4 h and it remains at about 385 mV after 21 h, which is comparable to previous studies [46].

The relationship between impedance data and frequency of complex capacitance model is essential to analyze the supercapacitor electrodes [28]. Figure 6c depicts the relationship between the real part of the capacitance $C^{\prime }\left(\omega \right)$ with the frequency of CNTs/CC, α-MnO_2_/CC, and α-MnO_2_@CNTs/CC, according to Equations S1, S2, and S3. The capacitance change is the one commonly described in the supporting information [47]: when the frequency decreases, $C^{\prime }\left(\omega \right)$ sharply increases, then tends to be less frequency-dependent and can be displayed by the change available of stored energy. This is characteristic of the electrode structure and the electrode/electrolyte interface and compares with CNTs/CC and α-MnO_2_/CC, while α-MnO_2_@CNTs/CC is more like an ideal capacitor [48]. As shown in Figure 6d and calculated from Equations (S1), (S2), and (S4), the time constants of CNTs/CC, α-MnO_2_/CC, and α-MnO_2_@CNTs/CC are 3.83 s, 2.15 s, and 1.21 s, respectively, so that α-MnO_2_@CNTs/CC shows a faster energy storage speed of nearly twice that of α-MnO_2_/CC and nearly three times that of CNTs/CC [47].

**Figure 7:**
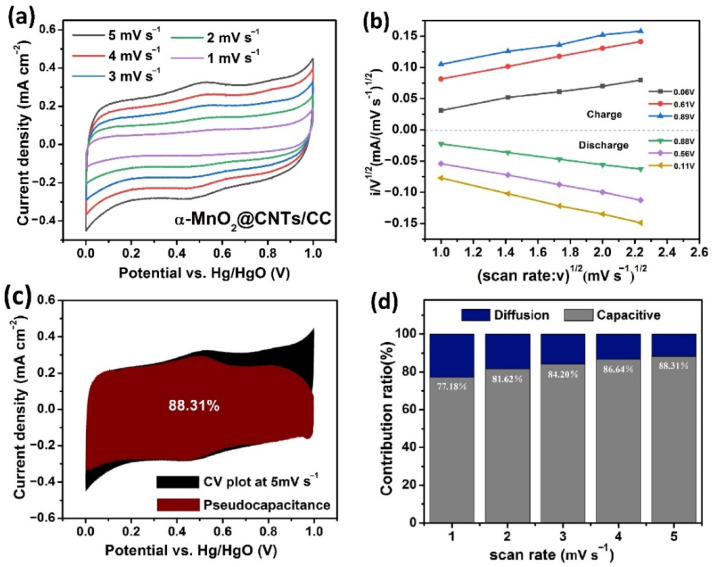
(**a**) CV curves of α-MnO2@CNTs/CC; (**b**) 6 selected sets of CV data obtained at different voltages for the calculation of k1 by Equation (4); (**c**) Pseudocapacitance of the α-MnO2@CNTs/CC electrode at 1 mV s−1; (**d**) Pseudocapacitance contribution ratios.

The mechanism of the α-MnO_2_@CNTs/CC electrode is explored by calculating the pseudocapacitance ratio based on the CV plots in Figure 7a and k1 (Figure 7b shows the images corresponding to six groups of random voltages during charge–discharge) [28] by Equation (6):

i(V)/v^1/2^ = k_1_v_1_/2 + k_2_, (6)

where i(V) represents the current at the selected voltage (V) according to the CV curves, v is the CV scanning rate, k_1_ is equal to the slope of the line obtained by fitting the connection points in the same voltage in Figure 7b, and k_1_v determines the current of pseudocapacitance at different potentials. The pseudocapacitance (red area) is obtained using k_1_v as the ordinate and the corresponding voltage as the abscissa, and the calculated Faraday pseudocapacitance accounts for the total capacitance (red area divided by the total CV area) from 1 mV s^−1^ to 5 mV s^−1^, as shown in Figure 7c,d. The calculated proportions of the pseudocapacitance are about 77.18% at 1 mV s^−1^ and 88.31% at 5 mV s^−1^, indicating that the pseudocapacitance makes the main contribution to the energy storage characteristics of α-MnO_2_@CNTs/CC. The pseudocapacitance contributions of α-MnO_2_/CC, derived from previous studies [28], show a 62.4% Faraday pseudocapacitance, accounting for the total capacitance at 1 mV s^−1^, which is lower than that of α-MnO_2_@CNTs/CC. The detailed pseudocapacitance contribution of the α-MnO_2_/CC electrode from 1 mV s^−1^ to 5 mV s^−1^ are summarized in Figure 4d of [28].

### 3.3. Electrochemical Performance of α-MnO_2_-Based Supercapacitor Devices with 1 M Na_2_SO_4_ Electrolyte

**Figure 8:**
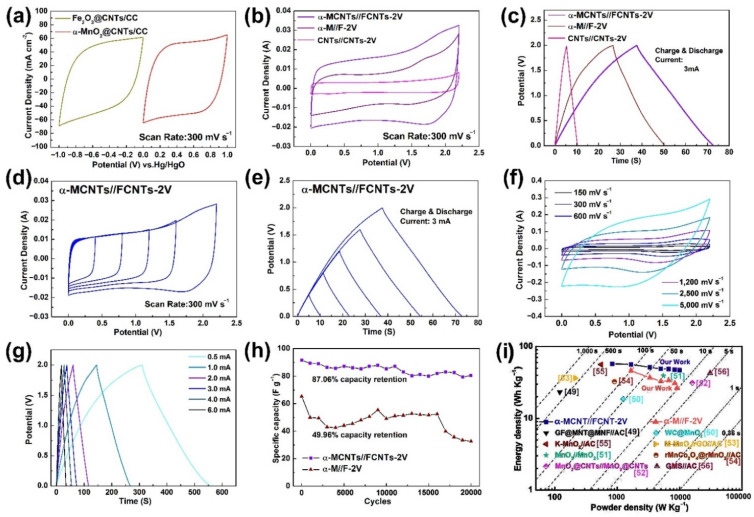
Properties of the α-MCNTs//FCNTs-2V supercapacitor in 1 M Na_2_SO_4_ electrolyte: (**a**) CV plots of α-MnO_2_@CNTs/CC and Fe_2_O_3_@CNTs/CC at 300 mV s^−1^; (**b**) CV plots and (**c**) GCD curves of α-MCNTs//FCNTs-2V, α-M//F-2V, and CNTs//CNTs-2V; Electrochemical properties of α-MCNTs//FCNTs-2V: (**d**) CV curves and (**e**) GCD curves of α-MCNTs//FCNTs-2V for different upper cut-off voltages, (**f**) CV curves, (**g**) GCD curves, (**h**) 20,000 cycles GCD test, and (**i**) Ragone plots showing the energy and power densities of the materials.

To assess the performance of the electrodes systematically, an asymmetrical supercapacitor is assembled with a 1 M Na_2_SO_4_ electrolyte, the positive electrode of α-MnO_2_@CNTs/CC, as well as a negative electrode of Fe_2_O_3_@CNTs/CC separated by a nonwoven fabric membrane. The supercapacitors of α-MnO_2_@CNTs/CC//Fe_2_O_3_@CNTs/CC (α-MCNTs//FCNTs-2V) and α-MnO_2_/CC//Fe_2_O_3_/CC (α-M//F-2V), shown in Figure 8 and Figure S5, are assembled in the CR2025 cell. The CV curves of the α-MnO_2_@CNTs/CC positive electrode and the Fe_2_O_3_@CNTs/CC negative electrode at 300 mV s^−1^ are drawn in Figure 8a for comparison. The two CV curves have a similar shape, area, voltage difference, and rapid response, corroborating the electrochemical properties and the same EDLC behavior. Figure 8b,c and Figure S6 show the electrochemical properties of the α-M//F-2V asymmetrical supercapacitor for comparison, and α-MCNTs//FCNTs-2V shows an obviously longer discharging time and larger CV area.

The CV and GCD curves of α-MCNTs//FCNTs-2V acquired at different voltage windows are presented in Figure 8d,e. In the 0–2.2 V window, both the CV and GCD curves do not exhibit obvious deformation, showing that the positive and negative electrodes match well to broaden the working potential of the device. With increasing scanning rates, the CV curves do not change, even at 5000 mV s^−1^, which is indicative of the rapid pseudocapacitive reaction on the electrode, as shown in Figure 8f. The GCD curves do not show much difference for the single electrode (Figure 8g), confirming the good electrochemical reversibility, pseudocapacitive characteristics, and I-V response. At a current of 0.5 mA, the specific capacitance is 103.27 F g^−1^, which is comparable to those of similar MnO_2_-based devices reported in the literature, as shown in Table S3. Figure 8h discloses that, even at a current of 10 mA, the asymmetrical supercapacitor has excellent cycling ability for 20,000 cycles, and it should be emphasized that such outstanding features are rarely observed from asymmetrical devices made of MnO_2_ and Fe_2_O_3_. Owing to the excellent properties of CNTs, the α-MCNTs//FCNTs-2V device retains 87.06% of its initial capacity, that is better than α-M//F-2V (49.46%) and most other devices shown in Table S3. According to Equations (2) and (3), the energy and power densities of α-MCNTs//FCNTs-2V are calculated and shown in Figure 8i and compared to other Mn-based supercapacitors in Figure 8i and Table S3 [49,50,51,52]. The total mass loading of α-MnO_2_ on CNTs/CC is about 0.73 mg cm^−2^ and the discharging time at different current densities are used to calculate the energy and power densities. At a power density of 833.35 W kg^−1^, the device has an energy density of 57.29 W h kg^−1^, and even at 9999.99 W kg^−1^, the energy density is still 46.95 Wh kg^−1^, which is better than those of M-MnO_2_/rGO//AC (36.4 W h kg^−1^ at 212.5 W kg^−1^) [53], rMnCo_2_O_4_@rMnO_2_-2 h//AC (32.4 W h kg^−1^ at 904.9 W kg^−1^) [54], K-MnO_2_//AC (56 W h kg^−1^ at 550 W kg^−1^) [55], and GMS//AC (42.77 W h kg^−1^ at 30,800 W kg^−1^) [56].

### 3.4. The Performance of 4 V Voltage Window α-MnO_2_-Based Flexible Supercapacitor with Ionic Liquid Electrolyte

**Figure 9:**
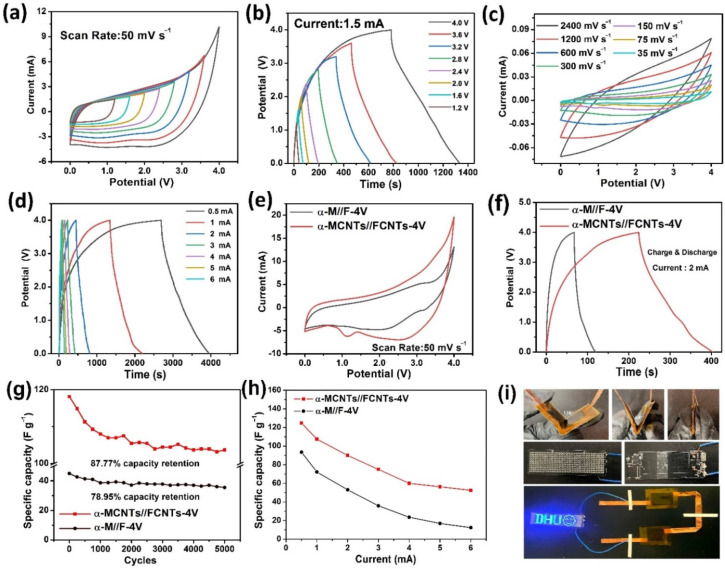
Electrochemical properties of the α-MCNTs//FCNTs-4V flexible supercapacitor in the EMImBF4 electrolyte: (**a**) CV plots and (**b**) GCD curves for different potential windows; (**c**) CV curves acquired at different scanning rates; (**d**) GCD plots obtained at different currents; Properties of the α-M//F-4V and α-MCNTs//FCNTs-4V flexible supercapacitor: (**e**) CV curves obtained at a scanning rate of 50 mV s^−1^, (**f**) GCD plots measured at a current of 2 mA, (**g**) 5000 cycles GCD evaluation, and (**h**) first cycle-specific capacitances measured at different currents; (**i**) Two assembled flexible ASCs in parallel powering the programmable LED arrays and with the ASCs bent at different angles.

To demonstrate the practical aspects, the ultrafast α-MCNTs//FCNTs-4V flexible supercapacitor with a large voltage window is fabricated, as described in Section 2.2 and shown in Figure 9. In the 0–4 V window, the GCD and CV curves do not exhibit obvious deformation, as shown in Figure 9a,b, confirming that the flexible supercapacitor can adapt to the broader 4 V window. The CV curves in Figure 9c indicate that the flexible device has good pseudocapacitive characteristics even at 2400 mV s^−1^, in addition to a fast I-V response with a good CV shape. The GCD curves are acquired up to 4 V at different currents (Figure 9d), and the discharging duration at the same current is more than three times that of the supercapacitor of α-MCNTs//FCNTs-2V in the aqueous electrolyte. Compared to the α-M/F-4V flexible supercapacitor in Figure S6, α-MCNTs//FCNTs-4V shows a larger CV area (Figure 9e) and a longer discharging time (Figure 9f) due to the carbon nanotubes. At a current of 0.5 mA, the discharging time of α-MCNTs//FCNTs-4V is 1261 s, whereas that of α-M//F-4V is only 200 s. Figure 9g discloses that the α-MCNTs//FCNTs-4V flexible device retains 87.77% of the initial specific capacitance after 5000 cycles at a GCD current of 2 mA, thus faring better than α-M//F-4V (78.95%) and most other devices listed in Table S4.

Figure 9h summarizes the corresponding specific capacitances of the α-MCNTs//FCNTs-4V and α-M//F-4V-based flexible device at different currents (from 0.5 to 6.0 mA^2^). The specific capacitances of α-MCNTs//FCNTs-4V are 124.8, 107.5, 90, 75, 60, 56.3, and 52.5 F g^−1^ at currents of 0.5, 1.0, 2.0, 3.0, 4.0, 5.0, and 6.0 mA, respectively. The electrochemical results of α-M//F-4V as the control are shown in Figure S6d, and the specific capacitances are 93.5, 72.2, 53.0, 35.9, 23.6, 13.5, and 10.8 F g^−1^ at currents of 0.5, 1.0, 2.0, 3.0, 4.0, 5.0, and 6.0 mA, respectively. Even though the charge current reaches 6 mA, the specific capacity of the CNTs-modified electrode retains 42.1% of the capacity at 0.5 mA. In comparison, for α-MnO_2_/CC//Fe_2_O_3_/CC, only 13.3% capacity is retained when the currents are changed from 0.5 mA to 6 mA, thus providing evidence that the flexible supercapacitor has good rate ability with the aid of CNTs. The energy densities of the α-MnO_2_@CNTs/CC-based flexible device are 166.7 Wh kg^−1^ at 3000.0 W kg^−1^ and 116.7 Wh kg^−1^ at 6000.0 W kg^−1^, which are superior to those of MnO_x_/N-rGOae in [BMP][DCA] + K_4_[Fe(CN)_6_], with an energy density of 44.68 Wh kg^−1^ at 1121.6 W kg^−1^ and a voltage window of 3 V [57]. A more detailed comparison is shown in Table 1, and the results confirm that α-MnO_2_@CNTs/CC with the EMImBF4 electrolyte produce high energy and power densities [58,59,60,61].

Figure S6 shows the Nyquist plots of α-M//F-4V before and after 5000 cycles. After 5000 cycles, the axial intercept increases from 6.32 Ω to 6.69 Ω. The radius of the semicircle in the first half of the impedance spectrum becomes larger, indicating that the specific capacitance decreases with the increasing charge transfer resistance. The slope of the straight line in the second half becomes smaller, implying that the charge diffusion resistance becomes smaller and the manganese dioxide structure collapses after charging and discharging. Figure 9i and Figure S7 show that the flexible supercapacitor can tolerate large mechanical bending at a large angle, and that different bending angles have little effect on the device performance. After charging at a high current for 10 s, two flexible devices in a series can light up the LED array consisting of electronic circuits and 11 × 44 small LEDs. The results unambiguously demonstrate that the ultrafast flexible supercapacitor has a wide voltage window and large commercial potential in energy storage systems.

## 4. Conclusions

Nanoscale MnO_2_ and Fe_2_O_3_ are fabricated on a conductive carbon fiber cloth modified with CNTs to form flexible electrodes for high-performance supercapacitors. The 2 V supercapacitor comprises the positive α-MnO_2_@CNTs/CC electrode and the negative Fe_2_O_3_@CNTs/CC in 1 M Na_2_SO_4_, whereas the 4 V ultrafast flexible supercapacitor uses the EMImBF4 electrolyte. The electrochemical characteristics and mechanisms in the different electrolytes are evaluated systematically. The results reveal the fast I-V response, outstanding pseudocapacitive characteristics, and excellent electrochemical reversibility due to the enhanced ion transfer efficiency between the electrodes and electrolytes, as well as the mitigated agglomeration of the nanomaterials. The α-MnO_2_@CNTs/CC and Fe_2_O_3_@CNTs/CC electrodes with excellent properties have a large potential in energy applications.

## Figures and Tables

**Table 1:** Comparison of the specific capacitances, energy densities, power densities, and capacitive retention of ionic liquid electrolyte-based supercapacitors.

Electrodes	Electrolytes	Potential Window (V)	Specific Capacitance (F g^−1^)	Energy Density (Wh kg^−1^)	Power Density (W kg^−1^)	Capacitive Retention	Refs
MnOx/N-rGOae	[BMP][DCA] + K4[Fe(CN)6]	3 V	144.45	44.68	1121.6	85.3% (after 20,000 cycles)	[57]
NiO/rGO	EMIBF4 + LiTFSI	4 V	56.7	146	1000	83.2% (after 4000 cycles)	[58]
Peanut-shell-derided AC	Mg(Tf)2 + EMITf	2 V	189	26	57,000	72% (after 10,000 cycles)	[59]
NF/CNT/Au/MnO_2_	[Bmim]PF6/DMF	3 V	-	67.5	593.8	-	[60]
Mn_3_O_4_ NDs@NG//APDC	EMIMBF4	4 V	56	124	999.3	82.4% (after 20,000 cycles)	[61]
α-MCNTs//FCNTs-4V	EMIMBF4	4 V	124.8	166.7	3000.0	87.77% (after 5000 cycles)	This work
α-M//F-4V	EMIMBF4	4 V	78.2	160.4	2000.0	78.95% (after 5000 cycles)	This work

## Data Availability

Not applicable.

## Supplementary Materials

The following supporting information can be downloaded at: https://www.mdpi.com/article/10.3390/nano12122020/s1, Figure S1. Electrochemical properties of α-MnO_2_/CC: (a) CV curves acquired at different scanning rates and (d) GCD curves obtained at different current densities; Figure S2. (a) CV curves obtained at a scanning rate of 600 mV s^−1^ and (b) GCD curves acquired at a current density of 0.25 mA cm^−2^ from CNTs/CC and Fe_2_O_3_@CNTs/CC; Electrochemical properties of CNTs/CC: (c) CV curves acquired at different scanning rates and (d) GCD curves obtained at different current densities; Figure S3. Specific capacitances of α-MnO_2_/CC and α-MnO_2_@CNTs/CC at different current densities; Figure S4. Electrochemical properties of the α-M//F-2V supercapacitor in 1 M Na_2_SO_4_ electrolyte: (a) CV curves acquired at a scanning rate of 300 mV s^−1^, (b) GCD curves obtained at a current of 3 mA with different upper cut-off voltages, (c) CV curves acquired at different scanning rates, and (d) GCD curves obtained at different currents; Electrochemical properties of CNTs/CC//CNTs/CC in 1 M Na_2_SO_4_ electrolyte: (e) CV curves acquired at different scanning rates and (f) GCD curves obtained at different currents; Figure S5. Electrochemical properties of the α-M//F-4V flexible supercapacitor in EMImBF4 electrolyte: (a) GCD curves, (b) CV curves for different upper cut-off voltages, (c) CV curves acquired at different scanning rates, and (d) GCD curves obtained at different currents; Figure S6. Nyquist plots of the flexible hybrid supercapacitor for the 4 V voltage window before and after 5000 cycles; Figure S7. (a) CV plots, (b) GCD curves, and (c) Nyquist plots with different bending angles; Table S1. Specific capacitances of α-MnO_2_/CC and α-MnO_2_@CNTs/CC in 1 M Na_2_SO_4_; Table S2. Important EIS parameters of the electrodes in Figure 5c,j; Table S3. Comparison of the electrochemical properties of MnO_2_-based supercapacitors. References [47,49,50,51,52,53,54,55,56,62] are cited in the supplementary materials.
