# Supplementary material for: Construction of α-MnO2 on Carbon Fibers Modified with Carbon Nanotubes for Ultrafast Flexible Supercapacitors in Ionic Liquid Electrolytes with Wide Voltage Windows
Source: Nanomaterials (Basel). 2022 Jun 11;12(12):2020. doi: 10.3390/nano12122020 (PMC9228112; doi:10.3390/nano12122020)
Supplement: Supplementary file 1 [file nanomaterials-12-02020-s001.zip › nanomaterials-1749302-supplementary.pdf]

nanomaterials

Supplementary Material

Construction of α-MnO_2 on Carbon Fibers Modified with Carbon Nanotubes for Ultrafast Flexible Supercapacitors in Ionic Liquid Electrolytes with Wide Voltage Windows

Mai Li [1,*], Kailan Zhu [1], Hanxue Zhao [1], Zheyi Meng [2,*], Chunrui Wang [1] and Paul K. Chu [3]

[1] College of Science, Donghua University, Shanghai 201620, China; 2202257@mail.dhu.edu.cn (K.Z.); z18238630680@163.com (H.Z.); crwang@dhu.edu.cn (C.W.)
[2] State Key Laboratory for Modification of Chemical Fibers and Polymer Materials, College of Materials Science, Donghua University, Shanghai 201620, China
[3] Department of Physics, City University of Hong Kong, Tat Chee Avenue, Kowloon, Hong Kong 999077, China; paul.chu@cityu.edu.hk
* Correspondence: limai@dhu.edu.cn (M.L.); mengzheyi@dhu.edu.cn (Z.M.)

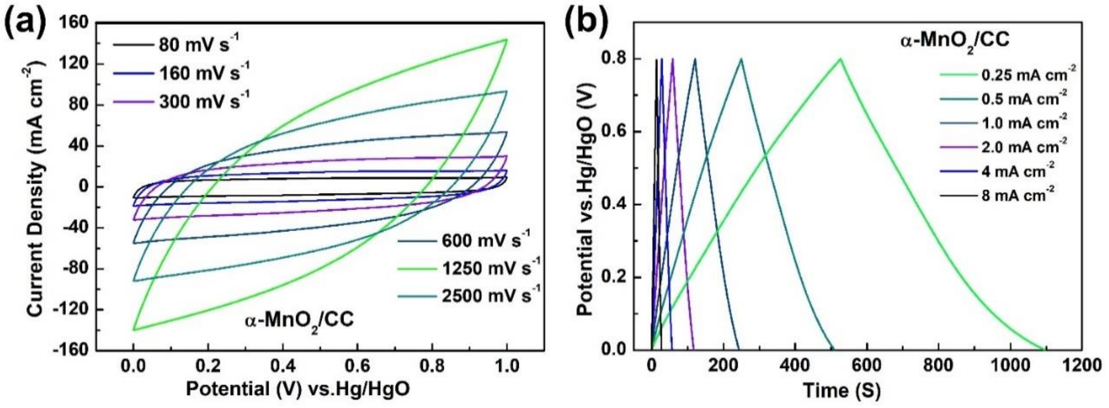

Figure S1. Electrochemical properties of α-MnO_2/CC: (**a**) CV curves acquired at different scanning rates and (**b**) GCD curves obtained at different current densities.

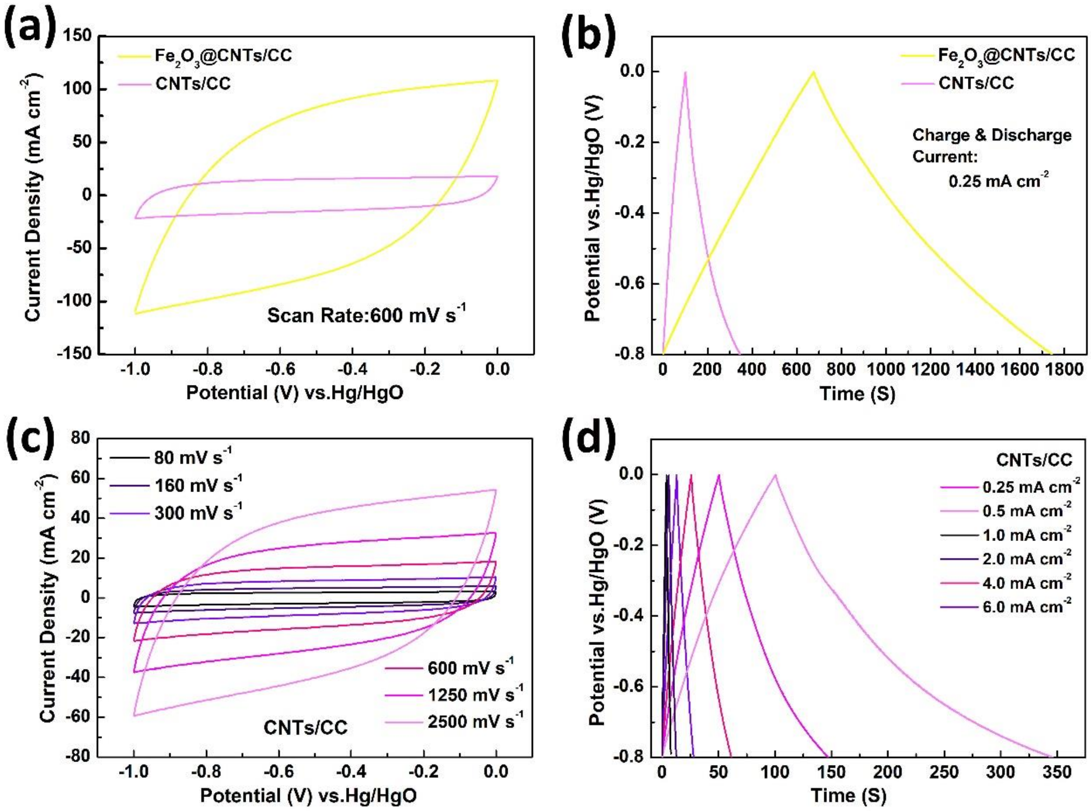

Figure S2. (**a**) CV curves obtained at a scanning rate of 600 mV s^{-1} and (**b**) GCD curves acquired at a current density of 0.25 mA cm^{-2} from CNTs/CC and Fe_2O_3@CNTs/CC; Electrochemical properties of CNTs/CC: (**c**) CV curves acquired at different scanning rates and (**d**) GCD curves obtained at different current densities.

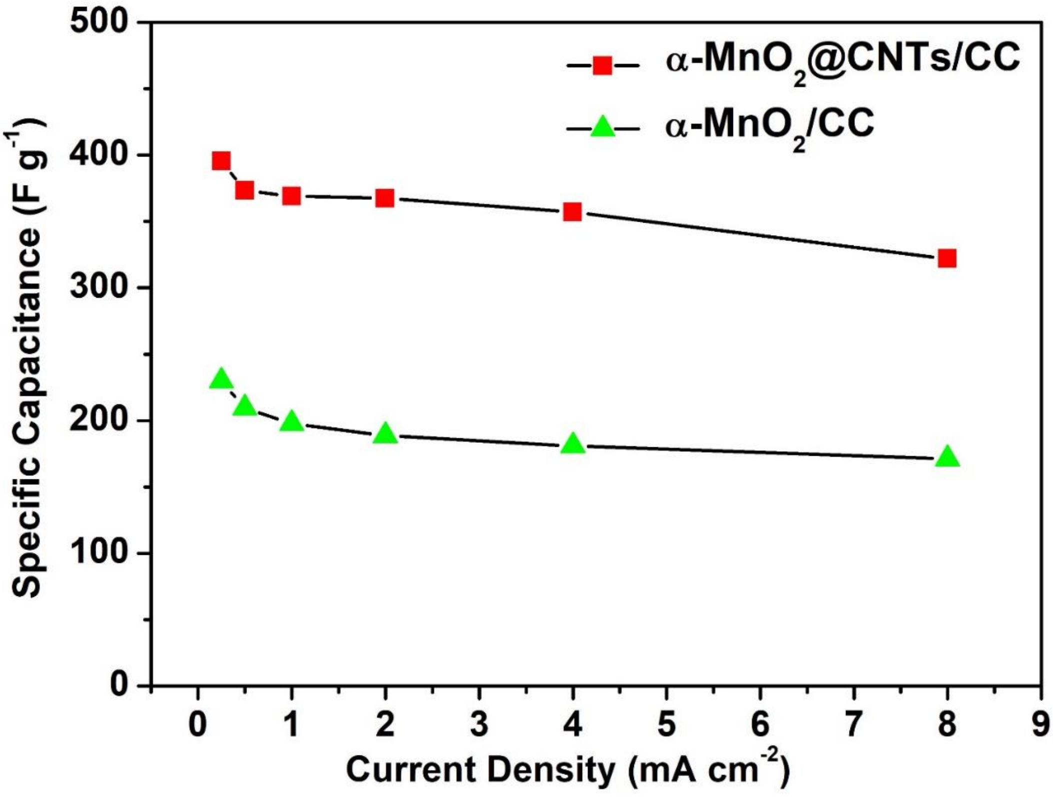

Figure S3. Specific capacitances of α-MnO_2/CC and α-MnO_2@CNTs/CC at different current densities.

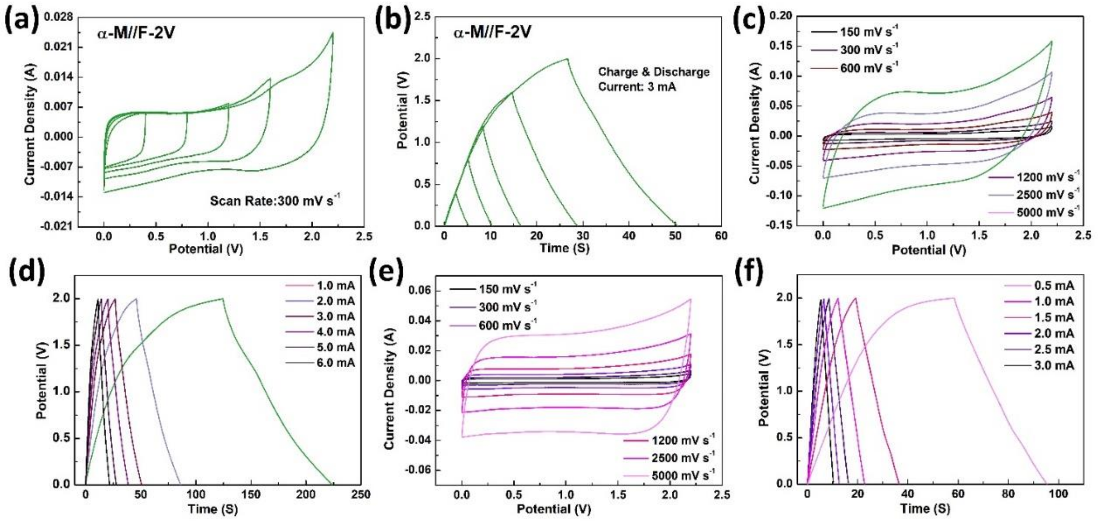

Figure S4. Electrochemical properties of the α-M//F-2V supercapacitor in 1 M Na_2SO_4 electrolyte: (**a**) CV curves acquired at a scanning rate of 300 mV s^{-1}, (**b**) GCD curves obtained at a current of 3 mA with different upper cut-off voltages, (**c**) CV curves acquired at different scanning rates, and (**d**) GCD curves obtained at different currents; Electrochemical properties of CNTs/CC//CNTs/CC in 1 M Na_2SO_4 electrolyte: (**e**) CV curves acquired at different scanning rates and (**f**) GCD curves obtained at different currents.

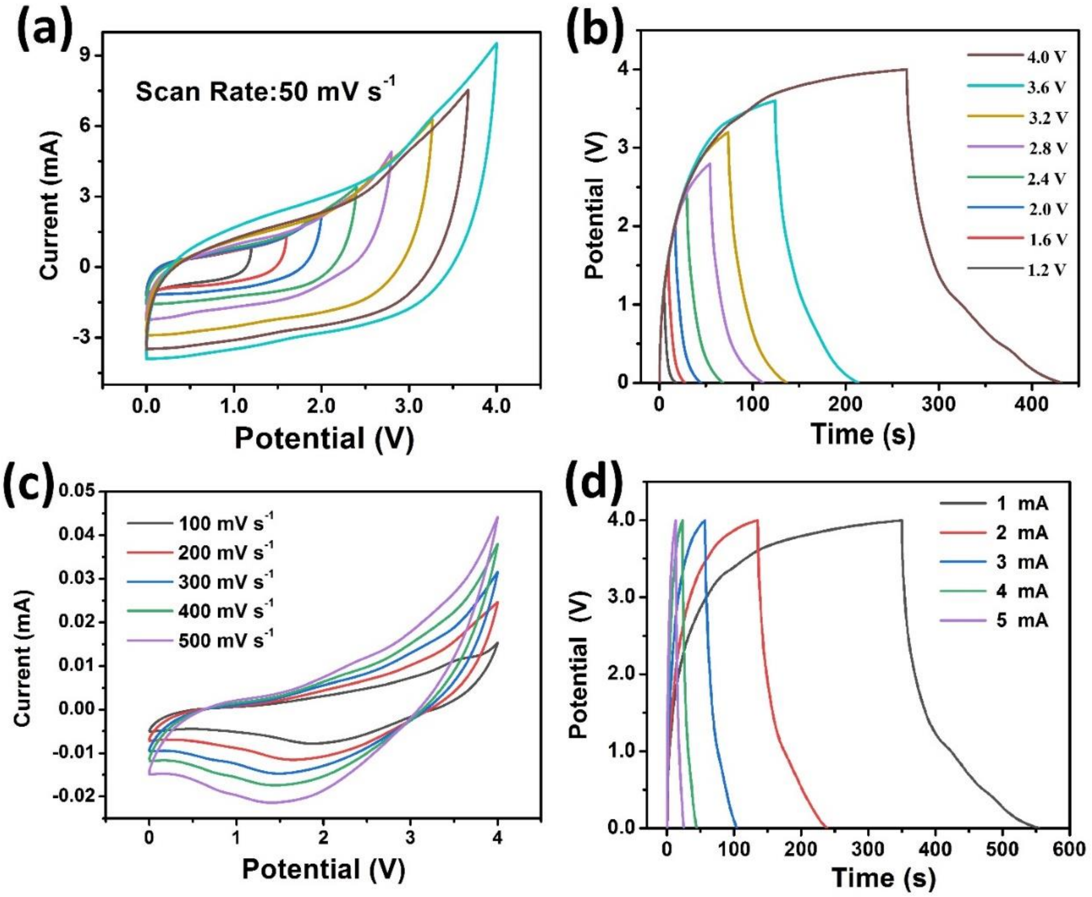

Figure S5. Electrochemical properties of the α-M//F-4V flexible supercapacitor in EMImBF4 electrolyte: (**a**) GCD curves, (**b**) CV curves for different upper cut-off voltages, (**c**) CV curves acquired at different scanning rates, and (**d**) GCD curves obtained at different currents.

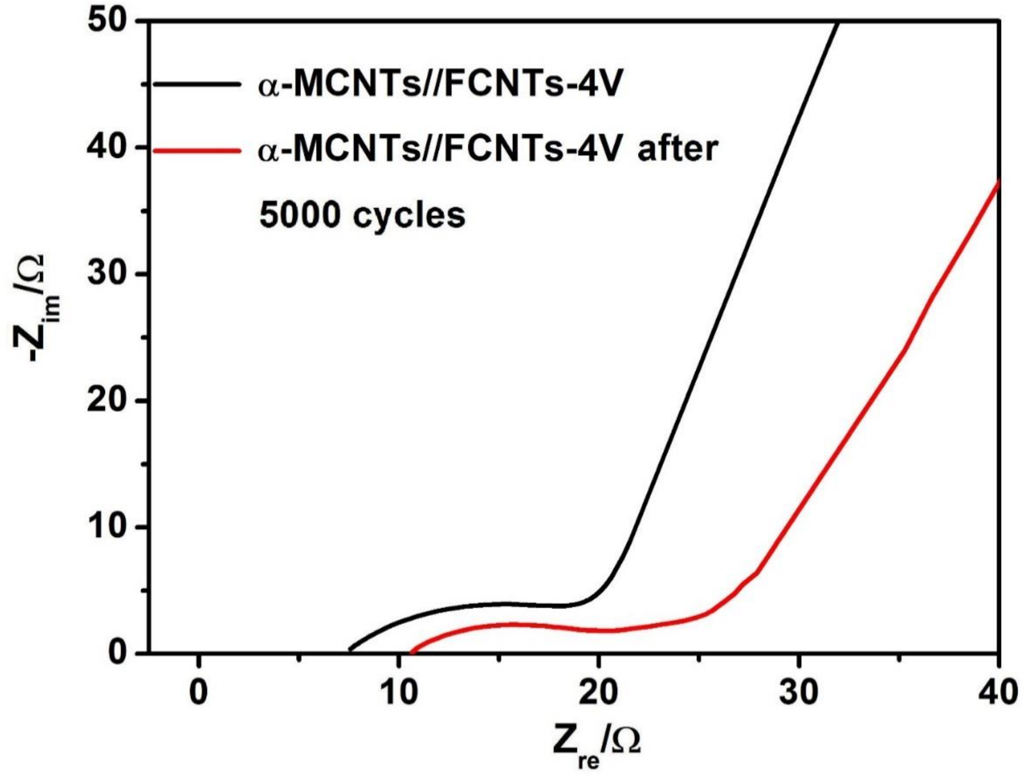

Figure S6. Nyquist plots of the flexible hybrid supercapacitor for the 4 V voltage window before and after 5000 cycles.

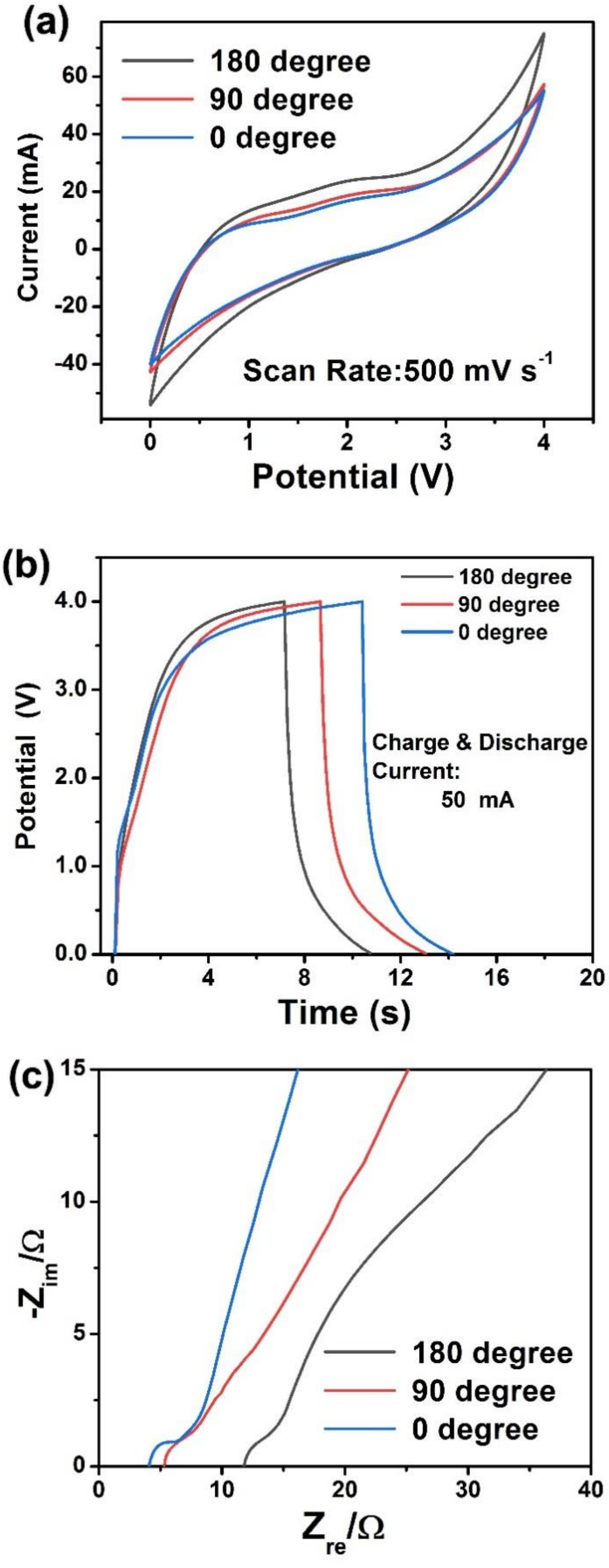

Figure S7. (**a**) CV plots, (**b**) GCD curves, and (**c**) Nyquist plots with different bending angles.

Real capacitance (C') and complex capacitance (C'') can be expressed by real, imaginary and total impedance with Equations (S1)–(S4), respectively [1,2]

$$Z(\omega) = \frac{1}{j\omega C(\omega)} \text{ in ohms} \tag{S1}$$

Copyright: © 2022 by the authors. Licensee MDPI, Basel, Switzerland. This article is an open access article distributed under the terms and conditions of the Creative Commons Attribution (CC BY) license (https://creativecommons.org/licenses/by/4.0/).

$$C(\omega) = C'(\omega) - jC''(\omega) \text{ in farads} \quad \text{(S2)}$$

Manipulation of equations (S1) and (S2) leads to

$$C'(\omega) = \frac{-Z''(\omega)}{\omega|Z(\omega)|^2} \text{ in ohms} \quad \text{(S3)}$$

$$C''(\omega) = \frac{Z'(\omega)}{\omega|Z(\omega)|^2} \text{ in ohms} \quad \text{(S4)}$$

where both $C'(\omega)$ and $C''(\omega)$ have units of farads F.

$C'(\omega)$ describes the real part of the capacitance and shows the function of frequency can be displayed by the change available of stored energy. $C''(\omega)$ describes the imaginary part of the capacitance and describes the energy loss corresponding to the form of energy dissipation [2].

Table S1. Specific capacitances of α-MnO_2/CC and α-MnO_2@CNTs/CC in 1 M Na_2SO_4.

Current Densities ($mA\ cm^{-2}$)	Specific Capacitance ($F\ g^{-1}$) α-MnO_2/CC	Specific Capacitance ($F\ g^{-1}$) α-MnO_2@CNTs/CC
**0.25**	229.65	395.35
**0.5**	209.30	373.23
**1**	197.72	369.17
**2**	188.55	367.44
**4**	180.822	357.01
**8**	171.17	321.75

Table S2. Important EIS parameters of the electrodes in Figures 5(c) and (j).

Electrodes	R_s	R_{ct}	CPE–P	CPE–T	W–R	W–T	W–P
**MnO_2@CNTs/CC**	4.32	1.16	0.40	0.031	0.014	8.94×10^{-5}	0.40
**MnO_2@/CC**	5.16	0.64	0.78	0.0016	0.0062	2.64×10^{-5}	0.37
**CNTs/CC (anode)**	5.12	4.87	0.52	0.0055	498.4	1.75	0.94
**Fe_2O_3@CNTs/CC**	5.60	0.30	1.14	2.90×10^{-5}	1.80	0.039	0.38
**Fe_2O_3@CNTs/CC after 10,000 cycles**	5.81	0.89	0.78	0.0014	42.21	3.40	0.69
**CNTs/CC (cathode)**	5.93	2.23	0.57	0.0031	0.020	2.3×10^{-5}	0.38

Table S3. Comparison of the electrochemical properties of MnO_2-based supercapacitors.

Electrodes	Electrolytes	Potential window (V)	Specific capacit y (F/g)	Energy density (Wh/kg)	Power density (W/kg)	Cycling retention (%)	Refs.
GF@MNT@MNF// AC	1 M Na_2SO_4	1.8	51.50	23.2	119.9	80% (5000–cycles)	[3]
WC@MnO_2// WC@MnO_2	6 M KOH	–	293.7	24.65	125	81.9% (5000–cycles)	[4]
K-MnO_2//AC	1M Na_2SO_4	2.2	83	56	550	98% (10,000–cycles)	[5]
M-MnO_2/rGO//AC	1M Na_2SO_4	~1.6	90.7	36.4	212.5	88.2%	[6]

						(10,000–cycles)	
MoO_2//MnO_2	1 M Na_2SO_4	2.2	58.6	39.4	5500	93.75 % (3000–cycles)	[7]
$rMnCo_2O_4$@$rMnO_2$-2h//AC	3 M KOH	1.6	91.2	32.4	904.9	81.8% (5000–cycles)	[8]
MnO_2@CNTs// MnO_2@CNTs	2 mM HTEMPO & 1 M Na_2SO_4	1.0	56	31.2	16,000	96% (10,000–cycles)	[9]
GMS//AC	1 M LiPF6 in 1:1 ethylene carbonate (EC)/dimethyl carbonate (DMC)	3.0	293.7	42.77	30,800	92% (10,000–cycles)	[10]
α-MCNTs//FCNTs-2V	1 M Na_2SO_4	2.0	103.27	57.29	833.35	87.06% (20,000–cycles)	This work
α-M//F-2V	1 M Na_2SO_4	2.0	82.92	46.06	1666.7	49.96% (20,000–cycles)	This work

References

1. Taberna, P.L.; Simon, P.; Fauvarque, J.F. Electrochemical characteristics and impedance spectroscopy studies of carbon-carbon supercapacitors. *J. Electrochem. Soc.* **2003**, *150*, A292–A300.
2. Soon, J. M.; Loh, K. P., Electrochemical Double-Layer Capacitance of MoS_2 Nanowall Films. *Electrochemical and Solid-State Letters* **2007,** 10, (11), A250–A254.
3. He, M.; Cao, L.; Li, W.; Chang, X.; Ren, Z. α-MnO_2 nanotube@δ-MnO_2 nanoflake hierarchical structure on three-dimensional graphene foam as a lightweight and free-standing supercapacitor electrode. *J. Alloys Compd.* **2021**, *865*, 158934.
4. Zhang, C.; Yu, X.; Chen, H.; Li, L.; Sun, D.; Chen, X.; Hao, X. Blocky woodceramics/nano-MnO_2 prepared by one-step hydrothermal activation as supercapacitor electrode. *J. Alloys Compd.* **2021**, *864*, 158685.
5. Zarshad, N.; Rahman, A.U.; Wu, J.; Ali, A.; Raziq, F.; Han, L.; Wang, P.; Li, G.; Ni, H. Enhanced energy density and wide potential window for K incorporated MnO_2@carbon cloth supercapacitor. *Chem. Eng. J.* **2021**, *415*, 128967.
6. Chen, L.; Yin, H.; Zhang, Y.; Xie, H. Facile Synthesis of Modified MnO_2/Reduced Graphene Oxide Nanocomposites and their Application in Supercapacitors. *Nano* **2020**, *15*, 2050099.
7. Zhao, C.; Hu, Y.; Zhou, Y.; Li, N.; Ding, Y.; Guo, J.; Zhao, C.; Yang, Y. Aerobic Recovered Carbon Fiber Support-Based MoO_2//MnO_2 Asymmetric Supercapacitor with a Widened Voltage Window. *Energy Fuels* **2021**, *35*, 6909–6920.
8. Liu, H.; Guo, Z.; Wang, S.; Xun, X.; Chen, D.; Lian, J. Reduced core-shell structured $MnCo_2O_4$@MnO_2 nanosheet arrays with oxygen vacancies grown on Ni foam for enhanced-performance supercapacitors. *J. Alloys Compd.* **2020**, *846*, 156504.
9. Zhang, Y.; Liu, Y.; Sun, Z.; Bai, Y.; Cheng, S.; Cui, P.; Zhang, J.; Su, Q.; Fu, J.; Xie, E. Strategic harmonization of surface charge distribution with tunable redox radical for high-performing MnO_2-based supercapacitor. *Electrochim. Acta* **2021**, *375*, 137979.
10. Rani, J.R.; Thangavel, R.; Kim, M.; Lee, Y.S.; Jang, J.-H. Ultra-High Energy Density Hybrid Supercapacitors Using MnO_2/Reduced Graphene Oxide Hybrid Nanoscrolls. *Nanomaterials* **2020**, *10*, 2049.
